# Molecular testing devices for on-site detection of *E. coli* in water samples

**DOI:** 10.1038/s41598-023-31208-4

**Published:** 2023-03-14

**Authors:** Carlos Manzanas, Elise Morrison, Young S. Kim, Morteza Alipanah, George Adedokun, Shouguang Jin, Todd Z. Osborne, Z. Hugh Fan

**Affiliations:** 1 Interdisciplinary Microsystems Group, Department of Mechanical and Aerospace Engineering, University of Florida, P.O. Box 116250, Gainesville, FL 32611 USA; 2 Department of Environmental Engineering Sciences, University of Florida, P.O. Box 116580, Gainesville, FL 32611 USA; 3 Department of Molecular Genetics and Microbiology, University of Florida, PO Box 100266, Gainesville, FL 32610 USA; 4 Whitney Laboratory of Marine Bioscience, University of Florida, P.O. Box 116580, St. Augustine, FL 32080 USA; 5 Soil, Water, and Ecosystem Sciences Department, University of Florida, P.O. Box 110290, Gainesville, FL 32611 USA; 6 J. Crayton Pruitt Family Department of Biomedical Engineering, University of Florida, P.O. Box 116131, Gainesville, FL 32611 USA

**Keywords:** Pathogens, Water microbiology, Diagnosis, Biomedical engineering, Mechanical engineering

## Abstract

*Escherichia coli* (*E. coli*) cells are present in fecal materials that can be the main source for disease‐causing agents in water. As a result, *E. coli* is recommended as a water quality indicator. We have developed an innovative platform to detect *E. coli* for monitoring water quality on-site by integrating paper-based sample preparation with nucleic acid isothermal amplification. The platform carries out bacterial lysis and DNA enrichment onto a paper pad through ball-based valves for fluid control, with no need of laboratory equipment, followed by loop-mediated isothermal amplification (LAMP) in a battery-operated coffee mug, and colorimetric detection. We have used the platform to detect *E. coli* in environmental water samples in about 1 h, with a limit of quantitation of 0.2 CFU/mL, and 3 copies per reaction. The platform was confirmed for detecting multiple *E. coli* strains, and for water samples of different salt concentrations. We validated the functions of the platform by analyzing recreational water samples collected near the Atlantic Ocean that contain different concentrations of salt and bacteria.

## Introduction

Water resources around the world are subjected to a variety of contaminants, either biological or nonbiological, and their presence beyond certain levels can be harmful to human beings^1^. Good public health requires regular water quality monitoring to prevent people from contracting diseases. Worldwide, approximately 1.6 million people die every year due to waterborne diseases caused by biological contaminants^1^, which affects countries of all economic levels^2^. Pathogens are the major biological contaminants in water and thus, it is important to monitor the presence of these in recreational and drinking water sources. Some of these pathogens include *Salmonella, Staphylococcus, Vibrio cholera*, *Legionella, Shigella, Escherichia coli (E. coli),* and other coliform bacteria^3^. These pathogens can be introduced to water sources and then enter the human body directly by drinking water or indirectly during bathing and other recreational water activities. For this reason, acceptable limits of some of these pathogens have been defined in legislation by different organizations such as the World Health Organization (WHO), the United States Environmental Protection Agency (EPA), or the European Union^4^.

Fecal pollution is the main source for disease‐causing agents in water^1,4^, including bacteria present in excreta from humans and warmed-blooded animals. *E. coli* is a type of bacteria that normally live in the intestines of warm-blooded animals, though some toxic strains (e.g., *E. coli* O157:H7) can cause abdominal cramps, vomiting, and diarrhea. Even small amounts of contaminated water with these toxic strains can cause illness^5^. Since humans and warm-blooded animals have *E. coli* present in their intestines, and these bacteria are released through feces, therefore *E. coli* can function as an indicator of fecal contamination in fresh water^4,6^.

The EPA reported an updated criteria in 2012 with a recommendation for fresh and marine water quality in recreational water^7^. They reported 2 criteria, one for an estimated illness rate (NGI, or NEEAR-GI illness while NEEAR stands for National Epidemiological and Environmental Assessment of Recreational Water and GI stands for gastrointestinal) of 36 per 1000 primary contact recreators, and one for 32 per 1000 primary contact recreators. The recommended criteria for *E. coli* in fresh water for the NGI of 32 per 1000 primary contact recreators in any 30-day interval is a geometric mean of 100 colony-forming units (CFU) per 100 mL and a statistical threshold value (STV) of 320 CFU per 100 mL^7^. As a result, the limit of quantitation (LoQ) of any method developed for water quality monitoring should be lower than 100 CFU/100 mL, or 1 CFU/mL.

Conventional methods for detecting pathogens in water are mostly culture-based approaches and separation/filtration techniques in laboratories^8^. Although these conventional laboratory assays are the standard methods due to their accuracy and sensitivity, they require bulky and costly instruments, skilled personnel, and long turnaround time. An extensively used testing system is the IDEXX Colilert, which can quantify the number of total coliform and *E. coli* in a single test. Although the IDEXX Colilert system is popular and relatively easy to use, it requires bulky and expensive equipment such as an incubator, with long time-to-result (24 h) due to bacterial culture^9^. Therefore, there is a growing trend to develop small, easy-to-use, and cost-effective devices for on-site methods for more rapid results^1^, which allow for immediate actions, potentially preventing people from contracting diseases. The development of low-cost, on-site methodologies can also benefit resource-limited countries or regions where laboratory settings are not available or easily accessible.

On-site portable platforms include approaches based on enzymatic assays^10–12^, microfluidics^13–15^, lateral flow strips^16,17^, and paper-based analytical devices^11,18^ coupled with fluorescence^19^, colorimetry^20^, or electrochemistry^21^ detection for rapid and easy interpretation of results. The often-cited limitations of these approaches include low sample volume processed (e.g., microfluidic devices), relatively low sensitivity due to no amplification (e.g., lateral flow strips and nucleic acid hybridization techniques), and long incubation times (e.g., enzymatic assays). On the other hand, nucleic acid amplification tests (NAAT) such as polymerase chain reaction (PCR) provide higher sensitivity and faster time-to-result than culture methods, and thus, they are often preferred^22–24^. Nevertheless, PCR approaches require sample preparation, sophisticated instrument, and trained personnel, while isothermal amplification techniques such as loop-mediated isothermal amplification (LAMP) are easier to implement due to no temperature-cycling requirement^25–27^. For example, Lee et al. employed syringe filters and magnetic beads to extract DNA, followed by LAMP in a portable instrument^27^.

In this work, we report the development of an on-site testing platform for *E. coli* detection in water. The platform can (1) process large sample volumes (1–10 mL) using a valve-enabled, paper-based sample preparation method, (2) amplify DNA using LAMP, and (3) detect amplicons based on color change. We used a 3D printed device and ball-based valves for sequential delivery of the reagents needed for cell lysis, DNA enrichment and purification, and the resultant DNA was concentrated onto a chromatography paper. LAMP was then achieved by a battery-powered, smart coffee mug that functions as a water bath, providing a constant temperature. SYBR Green dye was used for colorimetric detection. We have demonstrated detection of *E. coli* in environmental water samples in about 1 h (~ 30-min. sample preparation and ~ 45-min. LAMP) using this testing platform.

## Methods

### Device design and fabrication

**Figure 1:**
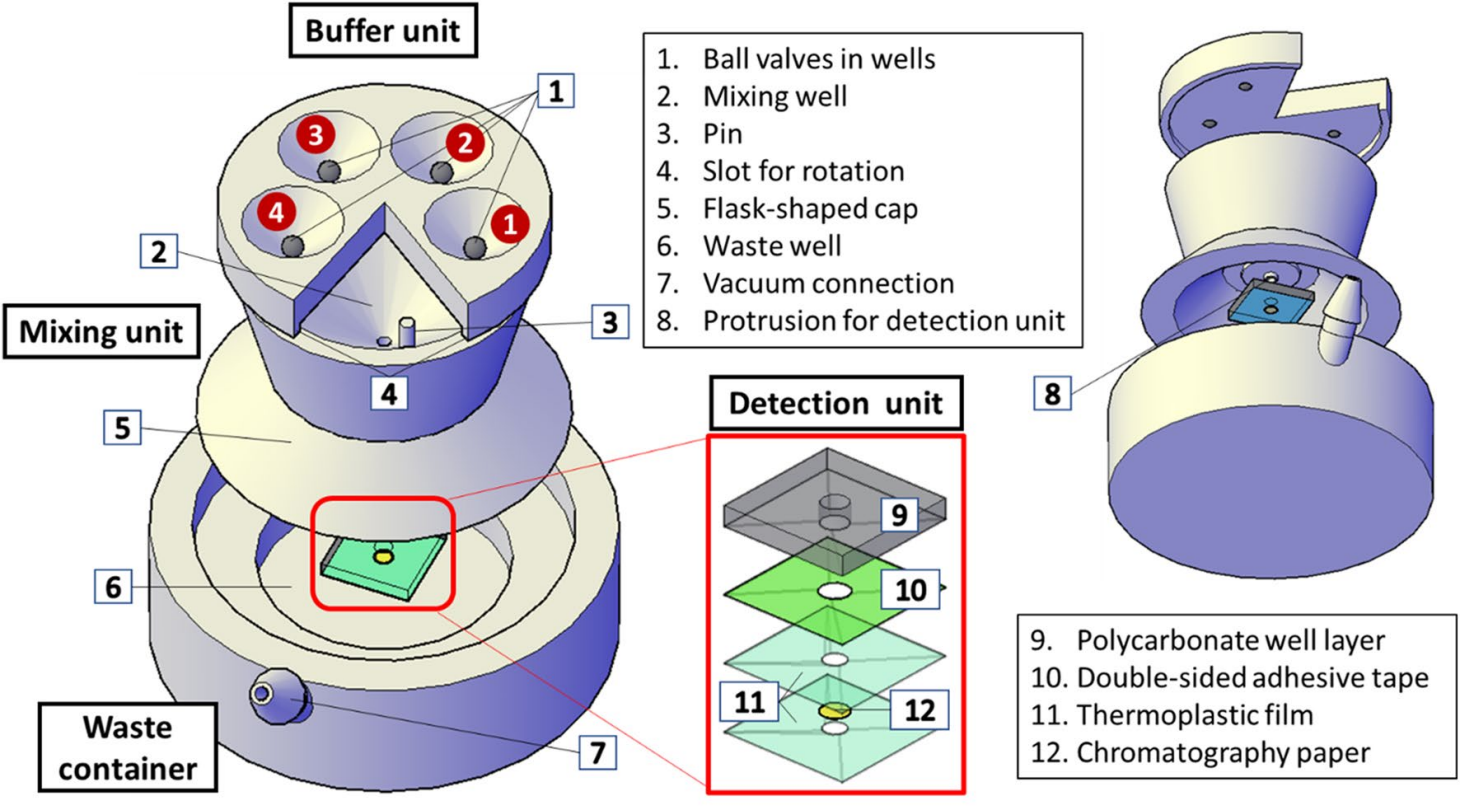
Exploded view of the device components. The buffer unit at the top contains four reservoirs and an opening. These 4 reservoirs are for the lysis buffer, binding buffer, and 2 wash buffers. The opening at the bottom of each well is blocked by a stainless-steel ball to prevent the reagents from flowing down until it is desired. The mixing unit in the middle is in a funnel shape to enhance mixing and make the reagents and sample pass through the paper in the detection unit, which is inserted by the protrusion (➇) at the bottom of the mixing unit. The mixing unit has a pin that pushes the ball up, opening the valve and allowing the reagents to go down when the buffer unit is rotated to align the pin with the ball. Finally, the waste container in the bottom serves 2 purposes, (1) collecting the waste, and (2) if needed, accelerating the filtration process by connecting a vacuum or a suction mechanism to it.

As shown in Fig. 1, the device consists of a buffer unit, a mixing unit, a detection unit, and a waste container. The detection unit was made of a polycarbonate well layer, double-sided adhesive tape, two layers of thermoplastic films, and a chromatography paper. The container was shaped into a 2 cm × 2 cm square from a 3-mm-thick polycarbonate sheet (McMaster-Carr, Elmhurst, IL) using a CNC milling machine (Sherline Products, Vista, California), and a well of 4-mm (or 6-mm) diameter was created in the center. One piece of Whatman™ 1 chromatography paper (Fisher Scientific) and two 75-μm-thick polyester thermal bonding lamination films (Lamination Plus, Kaysville, UT, USA) were cut into 3.5-mm or (5.5-mm) diameter circles using a Graphtec Craft Robo-S cutting plotter (Graphtec Corporation, Yokohama, Japan). The paper was then sandwiched between the two thermoplastic films and passed through a laminator, GBC® Catena 65 Roll Laminator (GBC, Lake Zurich, IL, USA), set at a rolling speed of “1” and at a temperature of 220°F as previously described^28,29^. This laminated paper pad was then attached to the polycarbonate well layer using double-sided adhesive tape (3 M 9087 white bonding tape, R. S. Hughes, Sunnyvale, CA), forming the detection unit.

**Figure 2:**
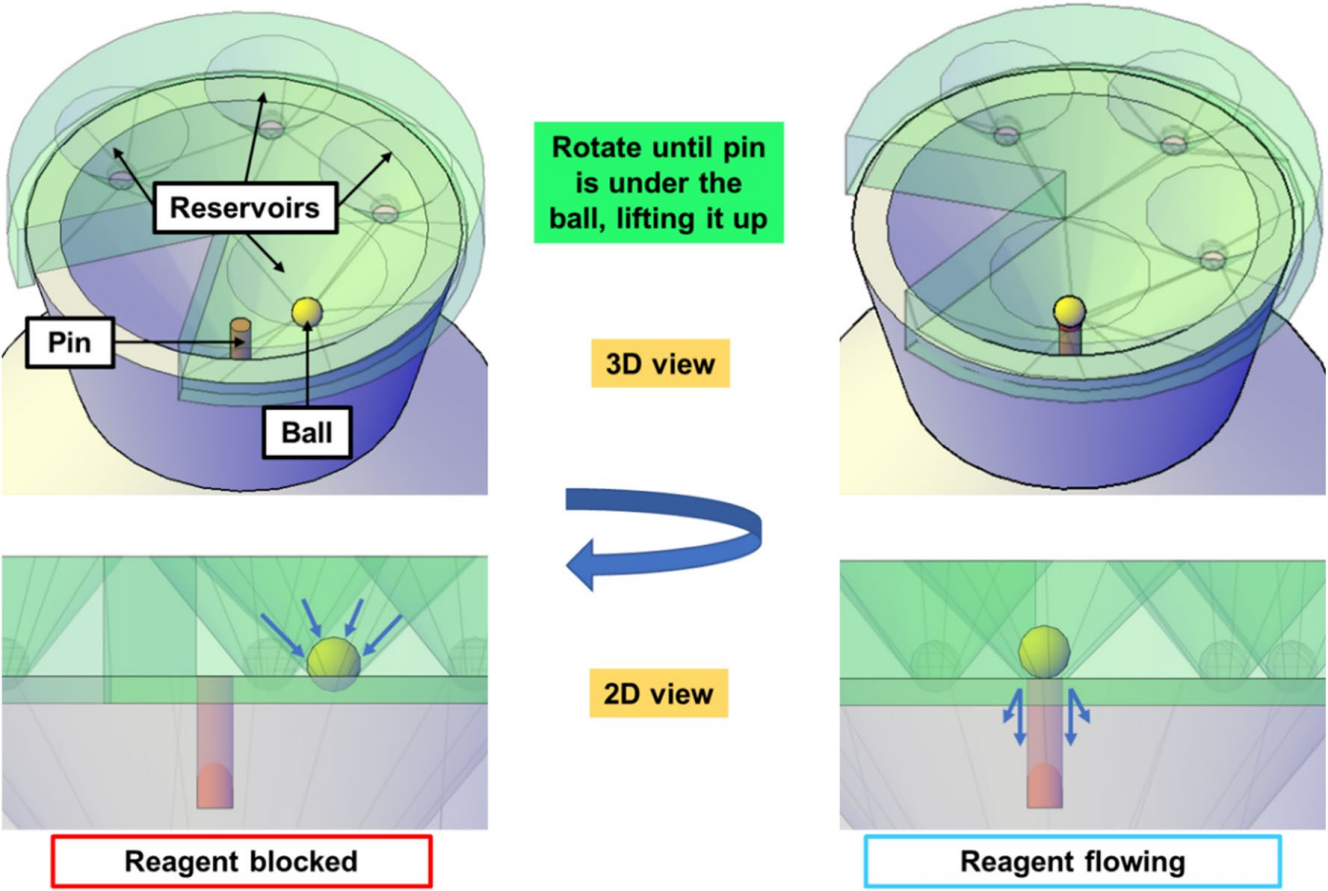
Ball-based valving mechanism. (left) Valve is closed when the ball blocks the reagent from flowing down. (right) Valve is opened after the buffer unit is rotated and the pin is aligned with the ball, lifting up the ball and allowing the reagents to flow into the mixing unit.

A commercial 3D printer, Ultimaker 3 (Ultimaker, Geldermalsen, Netherlands), was used to fabricate the buffer unit, the mixing unit, and the waste container. The devices were printed using acrylonitrile butadiene styrene (ABS), with the print layer height set to 0.1 mm and the infill density set to 100%. The balls used for valving in each well were 4.0-mm-diameter corrosion-resistant 316 stainless steel balls (McMaster-Carr, Elmhurst, IL). The valve concept is illustrated in Fig. 2. This valving mechanism is like a ballpoint pen, in which ink is dispensed onto paper when the metal ball at the tip of the pen is pressed while writing.

The buffer unit has a cylindrical shape with a diameter of 7.1 cm, a height of 1 cm, three reservoirs with a top diameter of 2 cm, and one with 2.4 cm, all with a depth of 1 cm, and a bottom diameter of 0.38 cm. The largest well ( in Fig. 1) is to accommodate the larger volume of a sample and reagent. These reservoirs can hold volumes up to 1.28 and 1.78 mL, respectively. The opening at the top was designed for 2 purposes: (1) visualizing when reagents go through the paper completely, moving onto the subsequent step, and (2) allowing, if necessary, for adding samples larger than 1 mL (up to 10 mL). The mixing unit has a height of 4.5 cm, an outside top diameter of 6.5 cm, an inside top diameter of 5.5 cm, an outside bottom diameter of 8 cm, and inside diameter of 6 cm. The wall thickness on the top is 1 cm, and 2 cm at the bottom, which helps achieve vacuum on the device without air leakage in the walls of the 3D printed material. The protrusion at the bottom of the mixing unit (part #8 in Fig. 1) allows it to fit snugly with the well of the detection unit. The liquid passage of the mixing unit to the detection unit has a diameter of 5 mm for integration with the 6-mm diameter detection units, which is located 3 cm from the top of the mixing well. The waste container has an outside diameter of 10 cm, and a height of 3 cm, however, these dimensions and those of the mixing unit could be reduced if no vacuum is used, or if a device is fabricated using a different manufacturing technique. Overall, when the device is assembled, the total height is 7.5 cm.

The sample preparation process of the device consists of sequentially releasing the buffer solutions for DNA lysis, binding, and washing from the buffer unit into the mixing unit through actuation of ball-based valves. These solutions are then directed to go through the detection unit for DNA enrichment and purification onto the chromatography paper. First, all buffer solutions are loaded into their respective reservoirs in the buffer unit, including 200 µL of AL lysis buffer (QIAGEN), 200 µL of ethanol, 500 µL of AW1 (QIAGEN), and 500 µL of AW2 (QIAGEN). Secondly, 1 mL of a water sample is added to the first reservoir containing the lysis solution to lyse the sample for 10 min, and then the mixture is discharged to the mixing unit by rotating the buffer unit and actuating the valve. This was followed by immediately rotating the buffer unit again to discharge the binding buffer from reservoir #2 to mix with the sample/lysis mixture. After these solutions completely go through the mixing unit and then into the paper pad in the detection unit, the buffer unit is rotated again to discharge the wash buffers, AW1 from reservoir #3, and AW2 from reservoir #4, one at a time to purify the collected DNA on the paper.

After DNA purification, the detection unit is detached from the mixing unit, ready for the next step: DNA amplification. After detachment, the bottom side of the detection unit is sealed using a piece of PCR tape, followed by adding the 50-µL LAMP mix using a disposable pipette. Another piece of PCR tape is put onto the top of the detection unit to seal the detection unit, preventing possible evaporation or leakage. The detection unit is then submerged in water in a coffee mug at 62.5 °C for 45 min. After amplification, the detection unit is taken out of the mug and the piece of tape at the top is removed, followed by adding 1 µL of SYBR Green for colorimetric detection. A schematic of the overall process and steps of the platform are shown in Supplementary Fig. S1. Supplementary Video S1 shows the entire process of the device from preparing the device until colorimetric detection of amplicons.

Fluid‐control valves are employed to perform sample preparation by sequential release of the reagents from the buffer unit into the mixing unit without the need of basic laboratory equipment. Each valve consists of a stainless-steel ball placed at the bottom of a buffer well to prevent the reagent from flowing down until it is desired. The ball protrudes 1.5 mm from the bottom of the buffer unit so that the pin in the mixing unit lifts the balls up and allows the reagents to flow down when the pin is aligned with the balls, as shown in Fig. 2. This valving mechanism is essentially the same as we reported previously^28,29^, though through horizontal sliding in the previous work, rather than rotation in this work. To prevent ball displacement and leakage during transportation, a breakable wax-based bond is created between the ball and the reservoir. First, a piece of wax (Akrowax™ 130, Akron, OH, USA) is heat-melted in a small beaker, followed by dipping a ball into the melted wax. The ball containing a thin layer of wax is immediately placed in the reservoir, allowing the wax to solidify and create a breakable bond to prevent any undesired movement.

### LAMP reaction

Each 25-μL LAMP mix contains 2.5 μL of 10× isothermal amplification buffer, 8 U Bst 2.0 WarmStart® DNA polymerase, 2.5 μL of 10× concentrated primer mix, and a final concentration of 1.4 mM dNTPs and 6 mM MgSO_4_. The 25-μL volume was filled up by nuclease-free water (not treated with diethylpyrocarbonate or DEPC). Except for the nuclease-free water and dNTPs from ThermoFisher Scientific (MA, USA), the other reagents in the LAMP mix were obtained from New England Biolabs (NEB) (Ipswich, MA, USA). The 10× primer mix contains 16 μM forward inner primer (FIP) and backward inner primer (BIP), 2 μM forward outer primer (often called F3) and backward outer primer (often called B3), and 8 μM loop primer forward (LF) and loop primer backward (LB); their sequences are shown in Supplementary Table S1. The primers were purchased from Integrated DNA Technologies (Coralville, Iowa, USA), and their sequences were chosen by following the literature^30^. When 50-μL LAMP mix was used to replace the 25-μL mix, the volumes of all reagents were doubled, keeping the same final concentration. To prevent non-specific amplification and possible false-positive results, uracil DNA glycosylase (UDG) and dUTP were added to the LAMP reactions to eliminate carryover contamination. UDG has been widely used to prevent carryover contamination without compromising sensitivity in LAMP and other nucleic acid amplification assays^31–33^.

To achieve LAMP without the need of connection to a power outlet, we chose a commercially available, battery-powered coffee mug (Ember™ Travel Mug, Ember Technologies, Inc., Westlake Village, CA) as a water bath as reported previously^28,29^. Prior to being placed in the Ember™ mug containing water at 62.5 °C, the detection units were sealed using two pieces of tape (Fellows®) to cover the bottom and top parts to prevent leakage and evaporation. After 45 min of incubation, the detection units were taken out for colorimetric detection, which was carried out by adding 1.0 μL of 10,000× concentrate SYBR green I in dimethyl sulfoxide (ThermoFisher Scientific) to each detection unit. We used SYBR green, a fluorescent dye, for colorimetric detection of amplicons; the color change can be visualized by the naked eye or recorded using a smartphone camera. To help visualization, an ULAKO blue LED (light-emitting diode) flashlight (Amazon, WA, USA) powered by one AA battery was used to observe the green fluorescence when *E. coli* were present. The results can also be verified by gel electrophoresis if needed. Note that LAMP produces a range of amplicons with different sizes; hence it does not have one specific gel band as with PCR^34^. We choose SYBR green since it detects the amplicons directly, while other dyes such as calcein^35^, hydroxy naphthol blue^36^ (HNB), or phenol red^37^ detect the by-products of LAMP amplification. Other dyes such as SYTO-9 are hard to visualize by the naked eye when they are at a low concentration that does not inhibit the LAMP reaction, but they can be used for real-time detection with an instrument^38^.

### LAMP time and sensitivity

To study the incubation time required for the LAMP assay, real-time LAMP experiments were carried out by adding 0.5 µL of 10X concentrate SYBR green I (ThermoFisher Scientific) to the 25-µL LAMP reaction mix. The fluorescence signal from the LAMP reaction was read through the QuantStudio 3 real-time PCR system (ThermoFisher Scientific). We tested different amounts of *E. coli* DNA, 8 × 10^4^, 8 × 10^3^, 8 × 10^2^, and 8 × 10^1^ copies, each of which was spiked into a 25-μL reaction solution. We tested 3 replicates of each concentration including 3 no-template controls (NTCs).

To assess the limit of quantitation (LoQ) of the LAMP assay for detection of *E. coli*, DNA was extracted from *E. coli* DH5-α cells using the QIAamp DNA Mini Kit (QIAGEN). The concentration of the purified DNA was determined to be 160 ng/µL using an ultraviolet–visible spectrophotometer. The copies/µL of the extracted DNA were calculated, which were determined to be approximately 3 × 10^7^ copies/µL using the molecular weight of *E. coli* genome. Serial dilutions of this stock solution were made using nuclease free water (Fisher Scientific). 1 µL of purified DNA of the different concentrations was added into 25-µL LAMP reactions, along with an NTC.

The number of copies were calculated based on the number of base pairs in the sequence, which was determined from NCBI GenBank (CP026085.1), containing 4,833,062 base pairs. Then, using the Eq. (1) below^39^, we calculated the number of copies of the purified DNA.

1 $${\text{Number of copies}} = \frac{{{\text{ng of double stranded DNA}} \times {\text{Avogadro}}'{\text{s constant}}}}{{{\text{number of base pairs}} \times 10^{9} \times 650\;{\text{Daltons}}}}$$

### Water sample testing

Environmental water samples were collected in the northeast Florida region, at the Pellicer Creek (map location: 29.66260 N 81.26837 W), Mouth of Pellicer Creek (29.66431 N 81.22892 W), and the Whitney Lab Docks (29.669249 N 81.216506 W). Pellicer Creek is linked to residential areas, and its water flow to or from (depending on tide) Whitney Lab Docks that is next to Atlantic Ocean. Sample #1 was collected from the Whitney Lab Docks on August 30, 2019, while sample #2 was collected from the Mouth of Pellicer Creek on September 6, 2019. Note that sample #1 was collected before Hurricane Dorian hit the area while sample #2 was collected after the hurricane. Both were filtered using a Whatman glass fiber filter and a peristaltic pump. Samples #3 and #4 (three replicates for each sample) were collected at the Pellicer Creek and the Whitney Lab Docks, respectively, on May 26, 2021. A summary of the environmental samples’ information is given in Supplementary Table S2. Samples #3 and #4 were received unfiltered and blinded by the researcher who performed the experiments to validate the platform. Some of samples #3 and #4 were filtered using a Whatman glass fiber filter (0.7 µm) and a 50 mL syringe, and the assay performance was compared between filtered and unfiltered samples. All water samples were processed using the device in Fig. 1.

### Effects of salinity

To study the possible effects of the salt in ocean water on sample preparation and LAMP assay and to demonstrate the capability of processing a wide variety of water samples, we tested the platform using samples prepared by spiking *E. coli* DH5-α cells into deionized (DI) water, containing 3.5%, 2.0%, 0.5%, and 0.0% (weight percentage) of sodium chloride (NaCl). These concentrations were chosen to simulate the concentration of salt in water from oceans (3.5%) to fresh water (~ 0%). Any concentration between them can be found in water at different points of estuaries. Five replicates were tested for each salt concentration. To prepare these samples, *E. coli* DH5-α cells in a suspension media were added to a 2-mL tube, centrifuged to form a cell pellet. After removing the supernatant media, the cell pellet was resuspended in 0.4 mL DI water and mixed by pulse-vortexing. The sample was then divided into four 2-mL tubes. Each tube was centrifuged again, and the DI water was discarded, followed by adding 1 mL of the water containing one type of salt concentration.

## Results

### Device design and fabrication

Figure 1 shows the design of the device for DNA extraction, enrichment, purification, and detection of *E. coli* in environmental water samples. The device consists of a buffer unit in the top, a mixing unit in the middle, a detection unit inserted on the bottom side of the mixing unit, and a waste container. The buffer unit contains the reagents needed for sample preparation; each solution is discharged by rotating the buffer unit over the mixing unit to actuate the ball-based valves. These fluid-control valves prevent the reagents from going down until the balls are lifted by the pin in the mixing unit. These reagents then mix and go through the detection unit for collection of purified DNA onto the chromatography paper, followed by DNA amplification via LAMP. The reagents can be pre-packaged in the buffer unit for storage and transportation while the wells are sealed, and the ball-based valves have shown no leakage when wax is used to fix the balls as shown previously^28^.

Our device integrates all the necessary steps for a nucleic acid assay, including lysis, DNA enrichment and purification, amplification, and detection. As a result, our platform eliminates the need for sample transportation from the sampling site to a lab. It also has much shorter assay time than culture-based approaches. Compared with traditional sample preparation methods based on solid extraction columns, the DNA enrichment onto a paper pad further reduces steps such as transferring DNA between tubes, avoiding possible contamination and degradation issues, and eliminating any elution step to extract the DNA from the solid column. The DNA in the paper pad in our device can be directly used for LAMP, offering significant advantages over the extraction column method because not all the DNA can be eluted from the column and there is no dilution when the elution step is eliminated. An untreated, cellulose chromatography paper was chosen for DNA enrichment after its comparison with commercially available FTA card and glass microfiber paper, showing slightly better nucleic acid enrichment from influenza viruses^40^.

### Effects of paper size

To optimize the paper size in the detection unit, we carried out the following analysis. We calculated the sample preparation time using Eq. (2). The volume of the sample is 1 mL, while the total volume of the reagents of lysis/binding/wash buffers is 1.4 mL. The flow velocity is 4.33 mm/min according to the manufacturer of the chromatography paper. It is understandable that the larger surface area of the paper circle is, the shorter the sample preparation time is. The calculated time as a function of the paper diameter is shown in Supplementary Fig. S2a. The results show that the theoretical preparation time is reduced from 44 to 19 min when the paper diameter is increased from 4 to 6 mm. We also observe that after a certain paper size (> 7 mm), the decrease in sample preparation time is not significant. At the same time, when the diameter of the paper circle increases, the volume of LAMP mix required to cover the whole area increases proportionally with the surface area (or square of diameter). Since the volume of the LAMP mix is 25 µL in a 4-mm well as we previously reported^28,29^, the volume of LAMP mix required for other paper circle size can be calculated using Eq. (3). The results are plotted in Supplementary Fig. S2b, and they indicate that the volume, and accordingly the cost of the LAMP mix, increases considerably. As a result, we chose a paper size of 6-mm diameter, and a LAMP mix volume of 50 µL for experimental comparison.

2 $$time \;\left( {min} \right) = \frac{{Volume\;of\;\left( {sample + reagents} \right)}}{Flow \;velocity*Surface \;area}$$

3 $$LAMP \;volume = 25 \upmu L* \frac{{D^{2} }}{{4^{2} }}$$

We used water samples #1 and #2 (both filtered when samples were provided) to compare the sample preparation time between the 4-mm and the 6-mm diameter paper pads. We saw a large difference as summarized in Supplementary Table S3. The samples took over 131 min. to process using the 4-mm diameter papers, whereas they took 23 min. for the 6-mm diameter papers. These results confirmed the much better performance of the larger paper size in our device. One downside of using a 6-mm diameter paper is its increase in the LAMP-mix volume as mentioned above, as well as resultant reagent cost. We also compared the sample preparation time of two additional types of samples using the 6-mm diameter papers: unfiltered water samples, and distilled water samples spiked with salts for the salinity experiments. We observed that the time decreased about 8 min. from the unfiltered samples (31 min.) to filtered samples (23 min.), and it was further reduced about 4 min from the filtered samples to the spiked ones (19 min.), respectively. The results are also summarized in Supplementary Table S3.

### LAMP time and sensitivity

**Figure 3:**
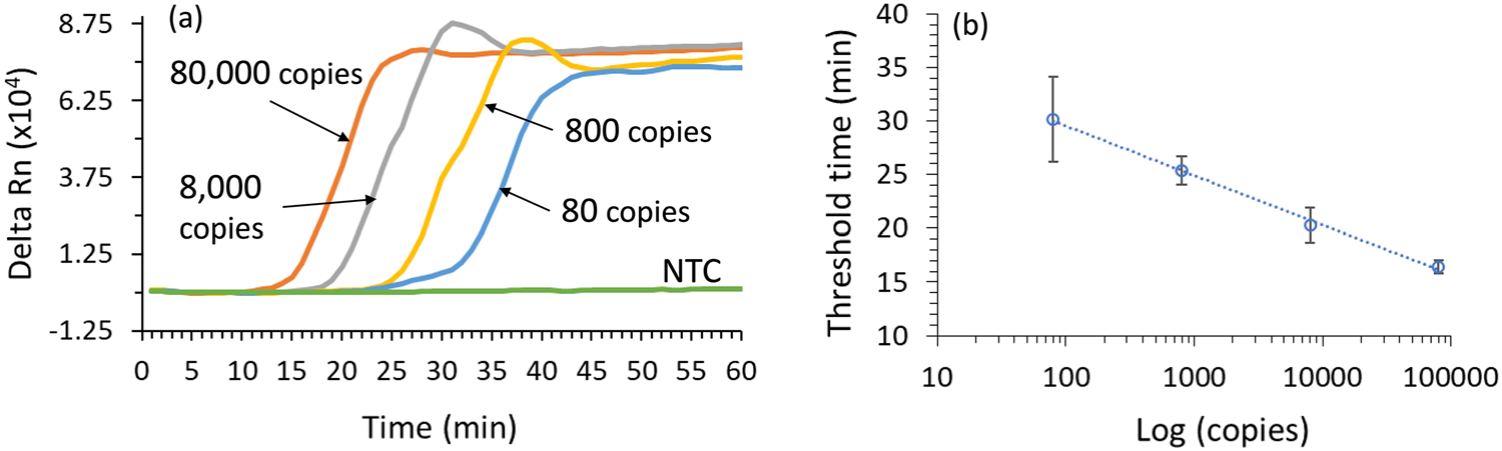
Real-time LAMP amplification using purified *E. coli* DH5-alpha DNA. (**a**) Fluorescent signal of 8 × 10^4^, 8 × 10^3^, 8 × 10^2^, and 8 × 10^1^ bacterial copies per reaction as a function of time. NTC, no-template control. (**b**) Calibration curve showing the threshold time (Ct) as a function of copies in each reaction (in log scale). The results were generated from 3 replicates of each concentration of DNA samples. The error bars indicate one standard deviation.

We employed a real-time PCR machine to identify the minimum time required for LAMP assay. Figure 3a shows the average fluorescence amplification curves for 4 different DNA concentrations, using 3 replicates for each curve. All the samples crossed the threshold line (defined as 10 times the standard deviation of the background noise above the baseline) within 35 min, and reached a plateau within 45 min. As a result, we chose 45 min as the LAMP reaction time. All reactions in Fig. 3 were incubated for 60 min, and no non-specific amplification was observed in all replicates of NTCs. Since LAMP involves many complex steps, the fluorescence signal is not correlated with the starting bacteria amount. However, the threshold time can be used to correlate with the initial amount of bacteria, as in PCR^41^. Figure 3b shows the calibration curve between the threshold time (that was provided by the instrument) and bacterial amount, indicating that semi-quantitative *E. coli* detection is feasible.

**Figure 4:**
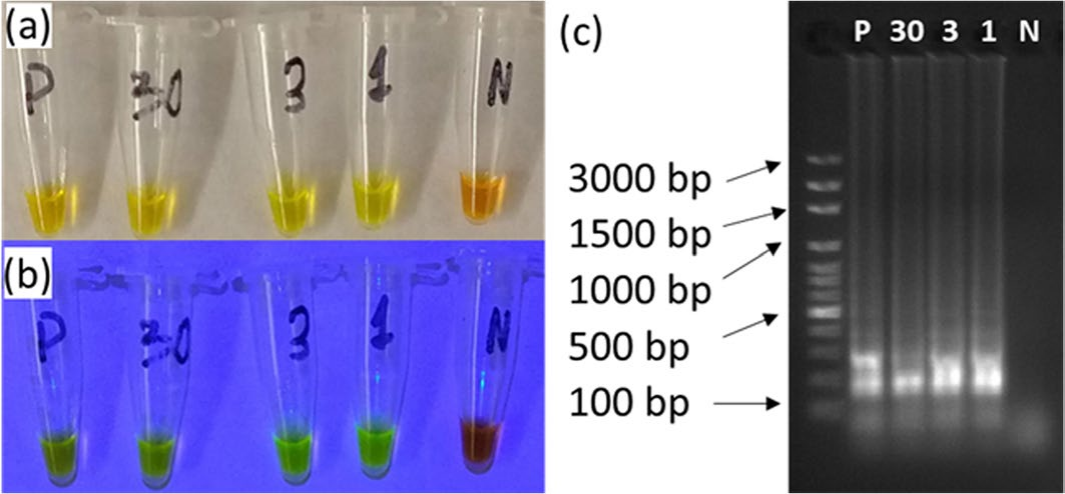
(**a**) Pictures of the reaction tubes taken under room lights after LAMP assay at 62.5 °C for 45 min. Amount of *E. coli* DNA are 300 copies for the positive control (P), and the others are marked on the tubes, 30, 3 copies, and 1 copy, as well as a negative control (N). (**b**) Same tubes of (**a**) under blue LED flashlight. (**c**) Gel electrophoresis of those samples in (**a**).

We studied the limit of quantitation of the LAMP assay using 300, 30, 3, and 1 copies of *E. coli* DH5-α DNA and observed the positive signals in all 5 replicates of 300, 30, and 3 copies as shown in Fig. 4. However, for 1 copy samples, we observed positive signals in only 1 out of 3 replicates, indicating the LoQ of our assay is between 3 and 30 copies, similar to the detection limit reported previously of 10 copies per reaction^30^. The results were confirmed using gel electrophoresis. The results for those repetitive experiments not contained in Fig. 4 are shown in Supplementary Fig. S3.

### Environmental water samples

We first tested the filtered water samples #1 and #2, whose total coliform were measured to be 517.2 CFU/100 mL and 270 CFU/100 mL using the IDEXX Colilert system. Note that total coliform is a collection of different types of bacteria, including *E. coli* and many others. After testing each sample five times, we obtained positive results for all five tests of sample #1, and four out of five tests for sample #2. Supplementary Figure S4 shows the results of 3 replicates for each sample.

Since the amount of *E. coli* in these two samples (#1 and #2) are not available (only total coliform was measured at that time), we collected more samples: three sample replicates from the Pellicer Creek (sample #3) and three samples from the Whitney Lab Docks (sample #4). After measuring the total coliforms and *E. coli* using the IDEXX Colilert system, these replicates were given as blind samples to the researchers who performed the experiments below using the device described in this work. First, each sample replicate was tested 2 times, and we got positive results from all 6 tests for three sample #4 replicates, but only 2 out of 6 tests for three sample #3 replicates, 1/2 for replicates #1 and #2, and 0/2 for replicate #3. To confirm the results, we tested sample #3 one more time, and got positive results for replicates #1 and #2, and negative for replicate #3.

**Figure 5:**
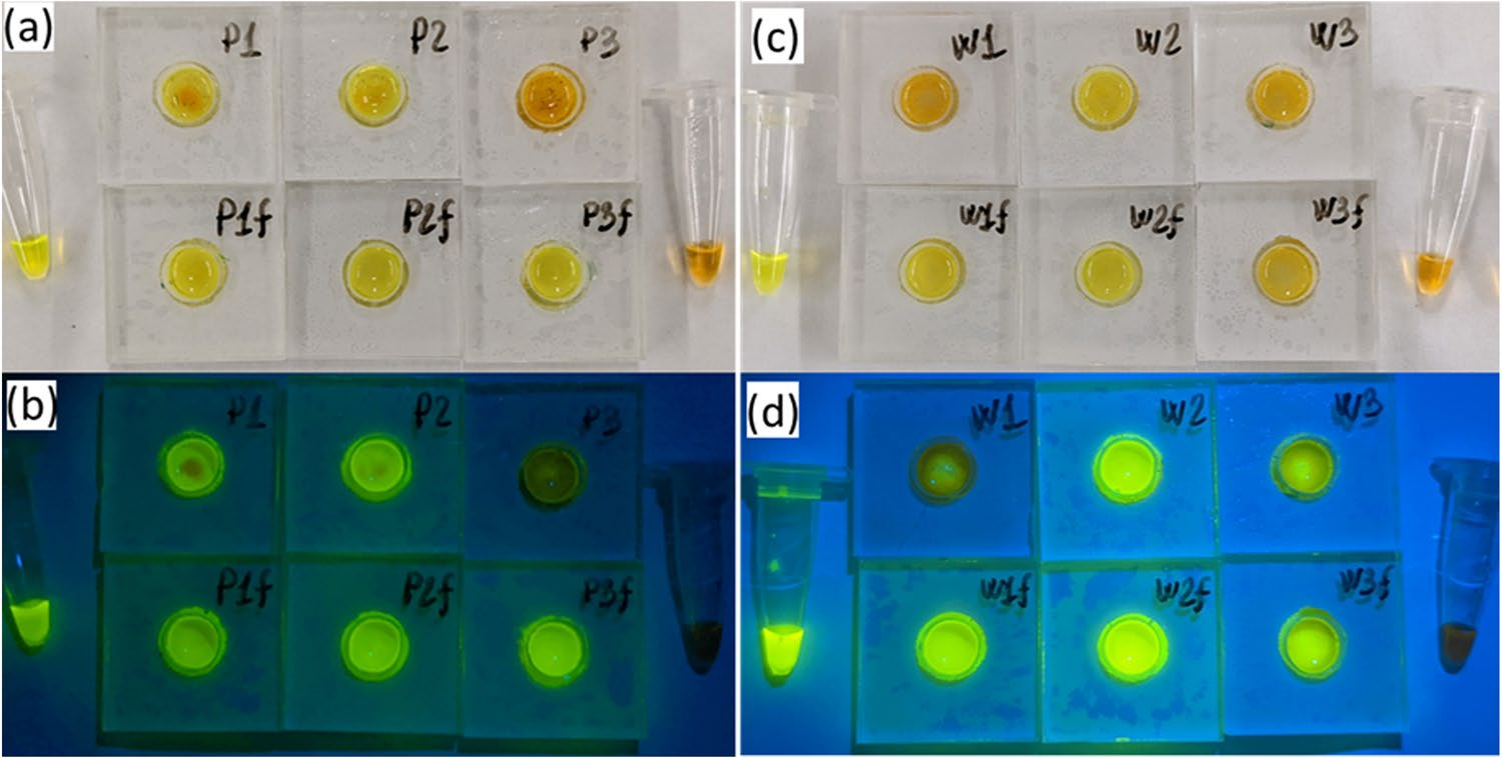
Pictures of detection units for sample #3 (**a**,**b**) collected at Pellicer Creek (P1, P2, P3 in for three sample replicates) and sample #4 (c and d) collected at Whitney Lab Docks (W1, W2, W3), and comparison between unfiltered (top row) and filtered (labeled with f, bottom row) samples, along with a positive control (left, in a tube), and a no-template control (right). Pictures in (**a**) and (**c**) were taken under room light while pictures in (**b**) and (**d**) were taken under a blue LED flashlight with a yellow plastic filter.

These test results were shared with those who provided blind samples and then compared with the total coliform and *E. coli* measured by the IDEXX Colilert system. Two sets of data did not correlate exactly with each other, as shown in Table 1; sample #3 had a higher *E. coli* concentration than sample #4 based on the IDEXX Colilert system whereas our device detected sample #4 more successfully than sample #3. During these experiments, it was noted that all sample #3 replicates, especially replicate #3, left numerous dark particles on the paper of the detection units. Thus, we suspected that some contaminants in sample #3 might have inhibited LAMP reactions. To verify, we filtered samples #3 and #4 using 0.7 µm Whatman glass fiber filters to remove the particulate materials just like sample #1 and #2 that were processed (i.e., previously filtered). Then, we tested the filtered samples, along with the unfiltered ones for comparison; these results are combined in Table 1 and some pictures of detection units are shown in Fig. 5.

**Table 1:** Test results of samples #3 and #4, and their comparison with total coliforms and *E. coli*.

Sample #	Sample #3						Sample #4					
Replicate #	1		2		3		1		2		3	
Total coliforms (CFU/100 mL)	> 2419.6		> 2419.6		> 2419.6		20		20		40	
*E. coli* (CFU/100 mL)	435.2		275.5		224.7		< 20		< 20		20	
Filtered (F) or unfiltered (U)	U	F	U	F	U	F	U	F	U	F	U	F
Positive tests*	4/6	3/3	5/6	3/3	0/6	3/3	3/4	3/3	4/4	2/3	4/4	3/3
Total per sample	9/18 (U)			9/9 (F)			11/12 (U)			8/9 (F)		

*The data are listed as the number of positive results/the number of experiments.

For sample #3, we had 100% success rate for filtered samples versus 50% success rate for unfiltered samples. These results confirmed that those particles filtered out of sample #3 inhibited LAMP reactions. For sample #4, the success rate between filtered and unfiltered samples is similar. The possible difference between sample #3 and sample #4 is that sample #3 was collected from Pellicer Creek which is linked to residential areas whereas sample #4 was collected from Whitney Lab Docks that is next to Atlantic Ocean. As a result, filtration during sampling as did for samples #1 and #2 should be employed for our device testing protocol to eliminate this concern. It should also be noted that filtration did not affect the detection as illustrated by sample #4, both filtered and unfiltered, with a similar testing success rate.

### Effects of salinity

To address the concern of possible effects of ocean water on the LAMP assay, we performed experiments using water samples spiked with various salt concentrations and the same amount of *E. coli* cells. The concentration of salt ranged from 0 to 3.5%, corresponding to fresh water and ocean water. For each salt concentration, we conducted 5 repeat experiments. For all salt concentrations, we obtained positive results in either 4 or 5 out of 5 tests, as summarized in Table 2**.** Supplementary Figure S5 shows the pictures of one representative testing result. These results suggest that the salt concentration at or below the level of the ocean water does not have a significant effect on either the sample preparation or the LAMP reactions. Note that three negative results in Table 2 took place in the first two experiments when mixing was not thorough enough before splitting and spiking *E. coli* cell into salt water. After addressing the issue, all replicates were successfully detected.

**Table 2:** Test results for the water samples spiked with various salt concentrations.

Salt concentration (wt%)	3.5%	2.0%	0.5%	0.0%
Positive tests*	5/5	4/5	4/5	4/5

*The data are listed as the number of positive results/the number of experiments.

### Limit of quantitation

Our platform with sample preparation and LAMP is sensitive, as indicated by consistent detection of *E. coli* in sample #4 (replicate #3), which contains 20 CFU/100 mL measured by the IDEXX Colilert system. This suggests the limit of quantitation (LoQ) of our platform is at least 0.2 CFU/mL, as we used 1 mL samples. Therefore, LoQ of our platform is at least five times lower than the threshold limit suggested by EPA, which is 100 CFU/100 mL^7^. Note that the LoQ of our LAMP assay itself was 3 copies using water samples spiked with *E. coli*, as shown in Fig. 4.

## Discussion

We have developed an on-site testing platform for *E. coli* detection in environmental water samples. Compared with most devices in the literature, one unique feature of our platform is its sample preparation, which consists of three simple steps: (1) rotating the buffer unit, (2) waiting for reagents to mix and go through the paper pad, and (3) removing the paper-based detection unit for subsequent amplification step. Amplification of the enriched DNA is achieved by immersing the detection unit inside a battery-powered coffee mug maintained at a constant temperature. The testing results are indicated by color changes, which can be observed by the naked eye, or recorded by a smartphone camera. Our platform is easy-to-use, portable for on-site testing, with low detection limit. Additionally, this platform can be easily adapted to detect other pathogens by simply modifying primer sequences in the LAMP assay.

The platform in this work follows similar valving concepts as in the previously developed VLEAD^28^ or 2-plex VLEAD^29^, but with significant modifications for the current application as summarized in Table 3. The main differences include (1) the operation mechanism that was changed from sliding to rotation, (2) the volume and type of sample processed, (3) the target pathogens, and (4) the kits and volumes of reagents used for the sample preparation process due to different pathogens/samples targeted. While VLEAD targets RNA viruses and captures their RNA, the device in this work targets DNA bacteria and captures their DNA. Because of the nature of the samples, the device in this work requires much larger sample volume since the *E. coli* present in recreational water is much less concentrated than viruses in human samples. This is also a part of reasons we added optional vacuum in this device to allow for faster time-to-result if any suction mechanism (e.g., syringes) is available at the point of testing. It is foreseeable to adapt this platform for wastewater-based studies such as those in estimating the prevalence and transmission of severe acute respiratory syndrome coronavirus 2 (SARS-CoV-2) in a community^42^.

**Table 3:** Comparison between VLEAD reported previously and this work.

	VLEAD	This work
Pathogens targeted	RNA viruses (Zika, SARS-CoV-2, influenza)	DNA bacteria (*E. coli*)
Molecular assay	RT-LAMP	LAMP
Time-to-result	50 min	75 min
Volume of samples	140 μL	1 mL
Type of samples	Bodily fluids or swap specimen	Recreational water
Operation mechanism	Sliding	Rotation
Volume of reagents (μL): lysis/binding/wash 1/wash 2	(QIAamp Viral RNA kit) 560/560/500/500	(QIAamp DNA kit) 200/200/500/500
Paper pad diameter	4 mm	6 mm
Possibility to add vacuum	No	Yes
Overall dimensions (L × W × H)	9 cm × 2.55 cm × 1.8 cm	10 cm × 10 cm × 7.5 cm

Our on-site, portable testing platform is able to (1) detect *E. coli* below the threshold limit of 100 CFU/100 mL using NAAT, (2) integrate all the necessary steps including sample preparation, DNA amplification, and detection into a single platform without the need of bulky or sophisticated laboratory equipment, and (3) obtain the results in about an hour. The reagents in our platform can be pre-packaged in the buffer unit for storage and transportation for on-site testing, and the ball-based valves have shown no leakage for several weeks when wax is used to fix the ball to the respective reservoir. The LAMP mix can also be pre-loaded in disposable pipettes and stored with ice packs. An alternative to cold storage is to use lyophilized RT-LAMP reagents that can be stored at room temperature as reported elsewhere^43,44^. Therefore, our platform can be used in the field, monitoring water quality on the spot.

Compared to other portable platforms for *E. coli* detection in water samples, our platform is one of the best in terms of time-to-result, LoQ, and simplicity, which are 3 important parameters for on-site testing platforms. Comparison of this work with other platforms for *E. coli* detection in water is shown in Table 4.

**Table 4:** Comparison of testing platforms for *E. coli* detection in water.

Refs.	Assay	Detection method	Lab equipment	Time-to-result	LoQ per mL	Input sample volume	Automated
^10^	Defined substrate technology	Absorbance and fluorescence	No	2–12 h	0.04 bacteria	25 mL	Yes
^11^	Paper-based culture	Luminescent and visual	For sample preparation	5.5 h	10 CFU	10 mL	No
^12^	Test strip + enzymatic substrate + culture	Color intensity	For sample preparation	5 h	0.5 bacteria	2 mL	No
^25^	Digital LAMP in membrane	Fluorescence	For assay, preparation, and detection	1 h	0.3–1000 cells	10 mL	No
^45^	Bead immuno-agglutination on paper	Mie scatter intensity—smartphone	For sample preparation	90 s	10 CFU	7 µL	No
^46^	Real-time PCR—microfluidic chip	Real-time PCR, fluorescence signal	For sample preparation and detection	~ 3 h	51 CFU	5 µL	Partial
^47^	LFA	Color change	No	1.5–3 h	200 CFU	Varies	No
^48^	Paper immuno-assay	Gold nanoparticles—scanner	For most steps	55 min	57 CFU	Few µL	No
This work	LAMP in paper-based unit	Color change	Disposable pipet	75 min	0.2 CFU	1 mL	No

One of the fundamental reasons for low LoQ of this platform is the use of a large water sample volume (1 mL). This sample volume compares favorably to other platforms, especially those based on microfluidics that can process only a few microliters^45,46^. Even if a device can detect a single cell, it is still not sufficient to detect lower concentrations than 100 CFU/100 mL (because there is no cell at all in 1 µL of water, unless a concentration procedure is carried out first). In addition, our platform offers higher sensitivity than paper-based immunoassays, which are not sufficient to detect concentrations below 100 CFU/100 mL, the threshold limit recommended by US EPA. The detection limit of paper-based immunoassays is mostly higher than 50 CFU/mL^47,48^.

Other approaches such as those based on enzymatic substrates offer high sensitivity and are capable of processing a large sample volume. However, they have long turnaround times, between 4 and 12 h of incubation, depending on the concentration of the samples^10–12^. Nucleic acid amplification assays offer both speedy analysis and high sensitivity. Lin et al. developed an asymmetric membrane to process up to 10 mL water samples, combining it with digital LAMP in the micropores of the membrane capable of detecting *E. coli* as low as 0.3 cells/mL within 1 h^25^. Nevertheless, it requires laboratory equipment for amplification and detection, including a fluorescence microscope^25^.

## Supplementary Information

Supplementary Information 1.

Supplementary Video 1.

## Data Availability

The data used during the current study will be made available from the corresponding author on reasonable request.
