# Supplementary material for: Molecular testing devices for on-site detection of E. coli in water samples
Source: Sci Rep. 2023 Mar 14;13:4245. doi: 10.1038/s41598-023-31208-4 (PMC10013241; doi:10.1038/s41598-023-31208-4)
Supplement: Supplementary file 1 — Supplementary Information 1. [file 41598_2023_31208_MOESM1_ESM.pdf]

Supplementary Information

Molecular Testing Devices for on-site Detection of *E. coli* in Water Samples

Carlos Manzanas[1], Elise Morrison[2,*], Young S. Kim[3], Morteza Alipanah[1], George Adedokun[1], Shouguang Jin[3], Todd Z. Osborne[4,5,*], and Z. Hugh Fan[1,6,*]

[1]Interdisciplinary Microsystem Group, Department of Mechanical and Aerospace Engineering, University of Florida, P.O. Box 116250, Gainesville, FL, 32611, USA

[2]Department of Environmental Engineering Sciences, University of Florida, P.O. Box 116580, Gainesville, FL, 32611, USA

[3]Department of Molecular Genetics and Microbiology, University of Florida, PO Box 100266, Gainesville, FL 32610, USA

[4]Whitney Laboratory of Marine Bioscience, University of Florida, P.O. Box 116580, St. Augustine, FL, 32080, USA

[5]Soil, Water, and Ecosystem Sciences Department, University of Florida, P.O. Box 110290, Gainesville, FL 32611, USA

[6]J. Crayton Pruitt Family Department of Biomedical Engineering, P.O. Box 116131, University of Florida, Gainesville, FL, 32611, USA

*Authors to whom correspondence should be addressed. e-mail: hfan@ufl.edu; elise.morrison@essie.ufl.edu; osbornet@ufl.edu

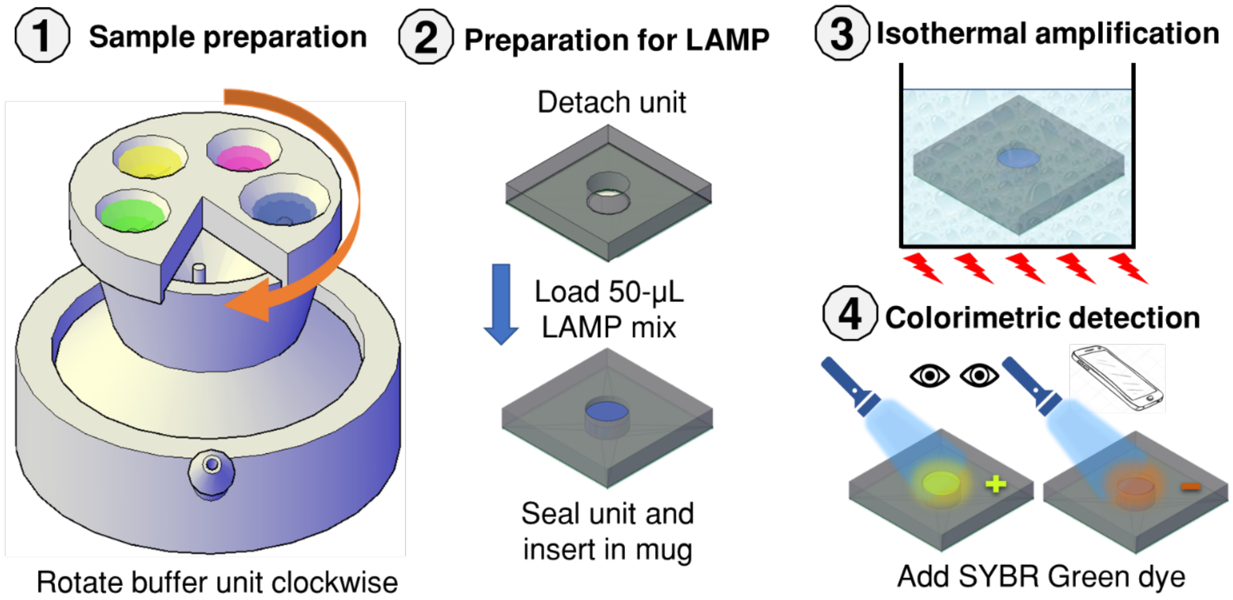

Figure S1. Workflow of the platform from the sample preparation until colorimetric detection.

Supplementary video S1 shows the device components, how to set up the device, the device operation for the sample preparation process, how LAMP is achieved, and how to determine the final results.

Table S1. Sequences of LAMP primers for *E. coli* detection targeting the malB region#.

**Primer name**	**Primer sequence (5’ - 3’)**	**Bases**
**F3**	GCCATCTCCTGATGACGC	18
**B3**	ATTTACCGCAGCCAGACG	18
**FIP (F1c + F2)**	CTGGGGCGAGGTCGTGGTATTCCGACAAACACCACGAATT	40
**BIP (B1 + B2c)**	CATTTTGCAGCTGTACGCTCGCAGCCCATCATGAATGTTGCT	42
**LF**	CTTTGTAACAACCTGTCATCGACA	24
**LB**	ATCAATCTCGATATCCATGAAGGTG	25

#Hill, J. *et al.* Loop-mediated isothermal amplification assay for rapid detection of common strains of Escherichia coli. *J Clin Microbiol* **46**, 2800-2804 (2008). https://doi.org:10.1128/JCM.00152-08. The paper stated that “the specificity of the assay was evaluated by testing eight gram-negative bacteria (*Acinetobacter* spp., *Citrobacter freundii*, *Enterobacter cloacae*, *Haemophilus influenzae*, *Klebsiella pneumoniae*, *Proteus mirabilis*, *Pseudomonas aeruginosa*, and *Serratia marcescens*) and six gram-positive bacteria (*Enterococcus faecalis*, *Staphylococcus aureus*, *Staphylococcus epidermidis*, *Streptococcus pneumoniae*, *Streptococcus pyogenes*, and *Streptococcus viridians*). No ladder pattern was seen with any of the other non-*E. coli* bacteria or with the negative “no-target” control.”

Table S2. Environmental water sample information.

Water Samples	Replicate #	Location	Collection Date
**#1**	1	Whitney Lab Docks (29.669249 N 81.216506 W)	August 30, 2019
**#2**	1	Mouth of Pellicer Creek (29.66431 N 81.22892 W)	September 6, 2019
**#3**	3	Pellicer Creek (29.66260 N 81.26837 W)	May 26, 2021
**#4**	3	Whitney Lab Docks (29.669249 N 81.216506 W)	May 26, 2021

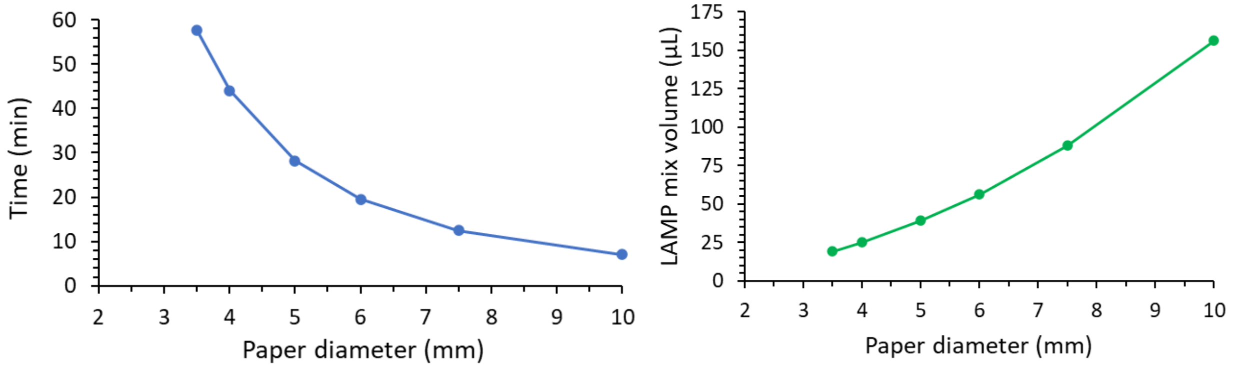

Figure S2. Sample preparation and LAMP mix volume needed as a function of the paper pad diameter. (a) Theoretical sample preparation time calculated for processing a 1-mL water sample through the chromatography paper as a function of the paper diameter, assuming a flow rate of 4.33 mm/min provided by the manufacturer of the paper. Equation 2 in the main text was used for calculation. (b) Volume calculated for the LAMP mix to cover whole area of a detection unit as a function of the paper diameter. Equation 3 in the main text was used for calculation and 25 µL mix in a detection unit with 4-mm diameter paper was used as a reference.

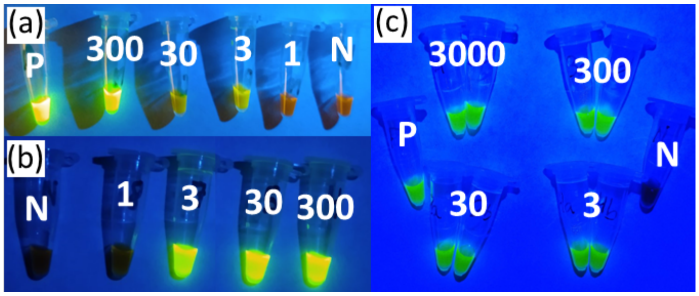

Figure S3. Pictures of the reaction tubes after LAMP assay, in addition to those in Figure 4 of the manuscript, for assessing the limit of quantitation. Amount of *E. coli* DNA are 300,000 copies for the positive control (P), and the others are marked on the tubes, 300, 30, 3 copies, and 1 copy, as well as negative control (N). (a) Experiment #1. (b) Experiment #2. (c) Experiment #3.

Table S3. Sample preparation time for different types of samples and paper sizes.

Paper diameter	***6 mm***			***4 mm***
Type of sample	*unfiltered*	*filtered*	*spiked*	*filtered*
Time (min)	31.22 ± 9.99	22.78 ± 5.45	19.39 ± 5.71	131.25 ± 55.49

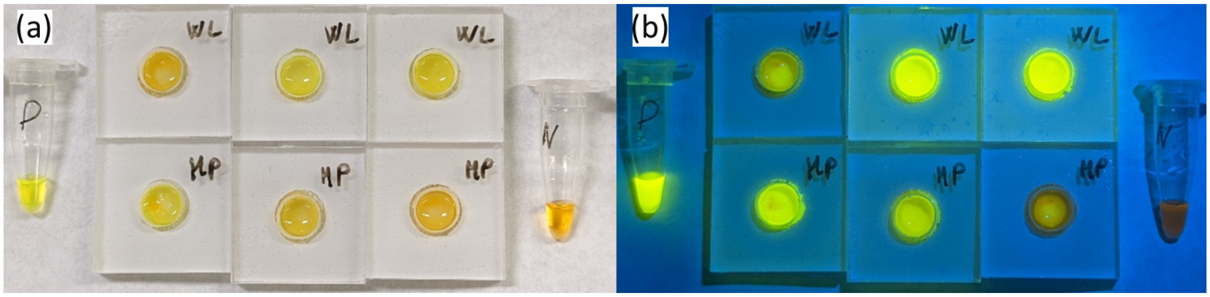

Figure S4. Pictures of the detection units in 3 replicates for analyzing sample #1 collected at Whitney Lab Docks (WL, top,) and sample #2 collected at Mouth of Pellicer Creek (MP, bottom), along with a positive control (P, left), and a no-template control (N, right). (a) Picture taken under room light; (b) Picture taken under blue LED flashlight with a yellow plastic filter.

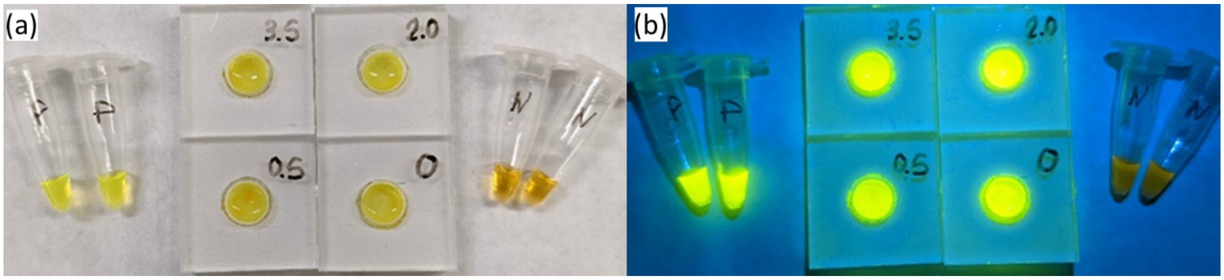

Figure S5. Pictures of detection units for analyzing the water samples spiked with 3.5% (top left), 2.0% (top right), 0.5% (bottom left), and 0.0% (bottom right) of salt concentration, along with two positive controls (P, left) and two no-template controls (N, right). (a) Pictures taken under room light; (b) Pictures taken under blue LED flashlight with a yellow plastic filter.
